# Group A rotavirus gastroenteritis: post-vaccine era, genotypes and zoonotic transmission

**DOI:** 10.1590/S1679-45082016RB3582

**Published:** 2016

**Authors:** Adriana Luchs, Maria do Carmo Sampaio Tavares Timenetsky

**Affiliations:** 1 Instituto Adolfo Lutz, São Paulo, SP, Brazil.

**Keywords:** Gastroenteritis/epidemiology, Diarrhea, Vaccination, Genotyping techniques

## Abstract

This article provides a review of immunity, diagnosis, and clinical aspects of rotavirus disease. It also informs about the changes in epidemiology of diarrheal disease and genetic diversity of circulating group A rotavirus strains following the introduction of vaccines. Group A rotavirus is the major pathogen causing gastroenteritis in animals. Its segmented RNA genome can lead to the emergence of new or unusual strains in human populations via interspecies transmission and/or reassortment events.

## RESUMO

Este artigo fornece uma revisão sobre imunidade, diagnóstico e aspectos clínicos da doença causada por rotavírus. Também aponta as principais mudanças no perfil epidemiológico da doença diarreica e na diversidade genética das cepas circulantes de rotavírus do grupo A, após a introdução vacinal. O rotavírus do grupo A é o principal patógeno associado à gastroenterite em animais. Seu genoma RNA segmentado pode levar ao surgimento de cepas novas ou incomuns na população humana, por meio de transmissão entre espécies e eventos de rearranjo.

## INTRODUCTION

Acute diarrheal disease (ADD) is a syndrome caused by different agents (bacteria, viruses and parasites), and its major manifestation is increased number of bowel movements, with watery or loose stools.^(1)^ Acute diarrheal disease or gastroenteritis is the most common disease all over the world and the main cause of death among children aged under five years.^(2)^ Acute diarrheal disease affects primarily children living in low- and middle-income countries, where the incidence rates are much higher specially due to poor quality of drinking water, inappropriate sanitation and nutritional risk factors, such as suboptimal breastfeeding, and zync and vitamin A defficiency.^(3)^ Brazil is a continent-sized country with much socioeconomic heterogeneity and the ADD monitoring data accounted a total of 33,397,413 reported cases, from 2000 to 2011 (http://portal.saude.gov.br). Data on total child mortality are published yearly, and the most recent estimate is of 2008. The estimated number of deaths due to ADD in children aged under 5 years was 1,336,289, in 2008, and Brazil accounted for 3,543 of these cases.^(2)^

Gastroenteritis in children is caused by a broad array of enteropathoegens; however, ADD is more often associated to rotavirus. This agent was described at least 40 years ago, and was soon recognized as the main cause of morbidity and mortalily associated to diarrhea.^(4)^ Virtually every child in both developed and developing countries will be infected by rotavirus in the first five years of life.^(5)^ All over the world, rotavirus ADD accounts for one third of 1,340,000 deaths, and for 9 million hospital admissions of children aged under 5 years.^(2)^

Rotavirus is also the main viral agent associated to gastroenteritis in animals, and was isolated in several species of domesticated and wild mammals,^(6,7)^ as well as in birds.^(8)^ These infections cause significant economic losses in livestock – cattle, swine and horses, because of cost of treatment and weight loss by affected animals. There is increasing evidence in the literature of interspecies transmission and reassortment between human and animal rotaviruses. Some species, such as dog, cat, pig and cattle, contribute in a more incisive and frequent manner to the genetic diversity found in humans.^(9)^

Therefore, the present article aims to present an overview on protective immunity, diagnosis, pathogenesis and clinical aspects of rotavirus ADD. It provides update information on vaccines against rotavirus that are available in the market, and informs about the main changes in epidemiology of gastroenteritis and genetic diversity of circulating strains in the post-vaccine era. Finally, it addresses the important interaction between human and animal rotaviruses.

## ROTAVIRUS STRUCTURE

**Figure 1:**
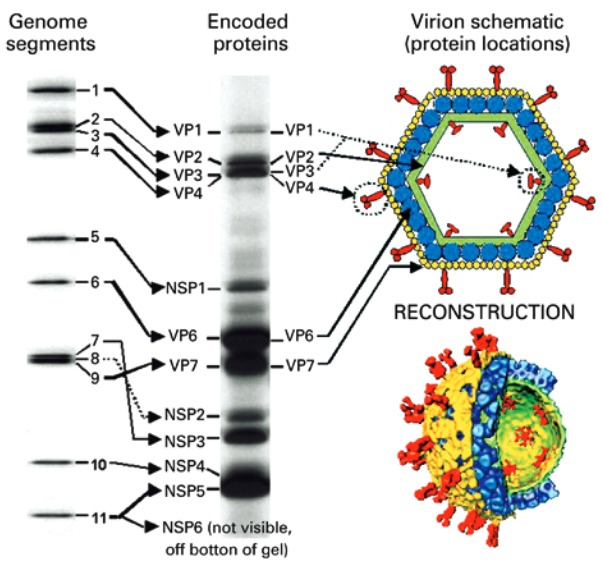
Schematic representation of the structure of a simian rotavirus particle (SA11). Note the correspondence between the double-stranded RNA segments (left), the schematic chart (top right) and the three-dimension structure of the virus on cryomicroscopy (bottom right)

VP: viral protein; NSP: non-structural protein*.*

Source: Mossel E, Estes M, Ramig F. Coding assignments and virion locations of rotavirus proteins and 3D structure of the rotavirus particle [Internet]. [cited 2015 Nov 23]. Available from: http://www.reoviridae.org/dsrna_virus_proteins/rotavirus%20figure.htm^(10)^

Rotaviruses belong to the Rotavirus gender and *Reoviridae* family*.* The whole viral particles are spherical, approximately 70-100nm in diameter, have an icosahedral capsid and no envelope. The capsid has three protein layers: inner, middle and outer capsid. The inner capsid or core has the viral genoma (Figure 1),^(1,10)^ which is composed of 11 double-stranded RNA segments (dsRNA). Each segment encodes a specific viral protein; in that, six structural proteins called viral proteins (VP) – VP1, VP2, VP3, VP4, VP6 and VP7, and six non-structural proteins (NSP) – NSP1, NSP2, NSP3, NSP4, NSP5 and NSP6. The rotavirus segments are monocistronic, except for segment 11 that encodes two proteins (NSP5 and NSP6).^(1,10)^

## PATHOGENESIS

Rotavirus has tropism for apical cells that line the small intestine villi, and infect the mature enterocytes. When disseminating in the enterocytes, rotavirus cause cell desquamation. The enterocytes are destroyed and migration of cells from the crypt to the cilli occurs faster, leading to temporary loss of the absorptive capacity of the intestine and to diarrhea.^(1,11)^ After cytolytic replication of rotavirus in mature enterocytes, the new viral particles released can also infect the most distal parts of the small intestine and/or be excreted through stools.^(12)^ The NSP4 protein plays a crucial role in the development of diarrhea by demonstrating functions of enterotoxin.^(1)^ Recent discoveries suggest that rotavirus infection can disseminate throughout the host body, leading to a systemic infection.^(13)^ Neurological manifestations associated to rotavirus infection have been reported and occur in approximately 2 to 5% of cases, ranging from benign seizures to lethal encephalitis. Nonetheless, it is still not clear if the rotavirus remains active and replicating in extraintestinal sites, or if the virus is just passively transferred through the bloodstream.^(11,13)^ Together, these data suggest pathogenesis of rotaviruses can be more complex than currently thought.

## ROTAVIRUS GROUPS

The Rotavirus gender comprises viruses that infect only vertebrates (birds and mammals).^(1)^ The rotaviruses have a common antigen – the protein VP6, which is present in the middle capsid (Figure 1),^(10)^ called group antigen.^(1)^ The group antigenic determinants conferred by VP6 enable classifying the rotavirus into five species, also called rotavirus groups, namely: Group A rotavirus (RVA), Group B rotavirus (RVB), Group C rotavirus (RVC), Group D rotavirus (RVD) and Group E rotavirus E (RVE) (http://ictvonline.org). There are three additional attempts of species: Group F rotavirus (RVF), Group G rotavirus (RVG) and Group H rotavirus (RVH).^(1,14)^ Recently, a new Group I rotavirus (RVI) was described in dogs.^(15)^

Group A rotavirus, RVB, RVC and RVH are associated to acute gastroenteritis in humans and animals. Group B rotavirus was detected in humans, cattle, sheep, pigs, does and rats. Group C rotavirus infects pigs, humans, cattle, dogs and ferrets.^(1)^ Group H rotavirus (J19, B219 and ADRV-N strains) was first detected in humans, in China and Bangladesh; more recentely, in pigs in Japan (SKA-1 strain) and Brazil (BR60, BR63 and BR59 strains).^(16,17)^ Group D rotavirus, RVE, RVF and RVG were only detected in animals.^(1,14)^ RVD, RVF and RVG affect exclusively birds.^(1,18)^ Group E rotavirus was detected only in pigs.^(9,19)^

## GROUP A ROTAVIRUS

Group A rotavirus is the group of relevance in terms of epidemiology and impact on public health, both in humans and animals,^(1,9,14)^ as is the core topic of this present review.

### Group A rotavirus classification

A binary classification system was established for RVA, based on immunological reactions and on the structure of VP7 and VP4 protein genes, which independently estimulate production of neutralizing antibodies (Figure 1).^(1,10,14)^ Therefore, the RVA strains are classified as VP4 or “P genotypes” (“P” refers to protease sensitive), and VP7 ou “G genotypes” (“G” refers to glycoprotein). So far, 27 G genotypes and 37 P genotypes have been described in RVA from humans and animals.^(14,20)^ In 2008, a classification system based on nucleotide analysis of the complete genoma was proposed for RVA. The system attributed a specific genotype for each of the 11 dsRNA segments, and the genes VP7-VP4-VP6-VP1-VP2-VP3-NSP1-NSP2-NSP3-NSP4-NSP5/6 of the RVA strains were described using the abbreviations Gx-P[x]-Ix-Rx-Cx-Mx-Ax-Nx- Tx-Ex-Hx (“x” represents the number starting from 1), respectively.^(14)^ The complete genoma sequence analysis of RVA significantly enhanced the capacity of recognizing the genetic relations between human and animal strains.

### Immunity against Group A rotavirus

The natural infection by RVA provides a significant clinical protection during an eventual reinfection. Cohort studies carried out in Mexico and Guinea-Bissau showed that recurrent episodes of RVA infection are considerably milder than the first event, resulting in protective efficacy against a second infection of 77 and 70%, respectively.^(21,22)^ However, this protection may not last longer, be incomplete or age-dependent.^(1)^

Primary and secondary infections caused by RVA are able to promote production of antibodies of the classes IgG, IgM and IgA in serum, saliva and intestinal secretions. VP6 is known as the most immunogenic protein, stimulating the production of IgA, primarily in the small intestine mucosa. The VP7 and VP4 proteins estimulate the production of serum neutralizing antibodies, conferring genotype-specific protection (homotypic) to hosts. Although the classic immunological response of RVA is homotypic, there is also a cross-reactive heterotypic response, with multiple genotypes. Reinfections by the same G or P genotypes may occur, thus corroborating the hypothesis of RVA incomplete protection. Passive immunity occurs by placental antibody transfer during pregnancy, and breastfeeding may protect neonates from RVA infection.^(1,23,24)^

### Clinical manifestations of Group A rotavirus

Group A rotavirus infection can result in a symptomatic or asymptomatic clinical picture, depending on virus and host factors (for instance, age or nutritional status). The estimated incubation period is 48 hours. The diarrheal episodes can vary from mild cases, with watery diarrhea and limited duration, to severe cases, including fever, vomiting, dehydration, electrolyte unbalance, shock and death.^(23)^ The onset usually manifests with fever (>39^o^C) and vomiting, and after 24 to 48 hours, watery diarrhea. The episodes of vomiting last less than 24 hours, and other gastrointestinal problems disappear within 3 to 7 days.^(25)^ Dehydration is a frequent complication, due to severity of diarrhea, associated to episodes of vomiting. Oral and/or intravenous rehydration therapy to maintain osmotic and electrolyte balance remain the basis to treat RVA infections.^(1)^

Immunocompromised children and adults (congenital immunodeficiency or organ transplants) infected by RVA can present prolonged diarrhea.^(25)^ However, there is no difference in terms of severity of diarrheal disease among children infected or not by HIV, even in Brazil.^(26)^

There are no specific antiviral agents against RVA infection. Rossignol et al.^(27)^ demonstrated nitazoxanide (brand name in Brazil is Annita^®^) may play an important role in control of viral gastroenteritis in adults, and showed good effectiveness against RVA. Kim et al.^(28)^ reported triacsin C analogues can act as potent antivirals in fighting RVA. Recently, Brazilian medicinal plants were tested *in vitro*. Among the species studied, *Byrsonima verbascifolia*, *Eugenia dysenterica*, *Hymenaea courbaril* and *Myracrodruon urundeuva* presented potential antiviral activity against RVA infection.^(29)^

### Vaccines against Group A rotavirus

In 1998, the Advisory Committee on Immunization Practices (ACIP) recomended the use of vaccine RotaShield^®^ (RRV-TV) (Wyeth Lederle Vaccines and Pediatrics, Marietta, Pennsylvania) in children, in the United States. This vaccine is composed of 11 simian genes (RRV) associated to human G1, G2 and G4 genotypes (gene VP7). In the clinical trials carried out in the United States, Finland and Venezuela, RRV-TV showed to be highly efficacious, significantly decreasing ADD caused by RVA in 80 to 100% of cases. However, in 1999, this vaccine was withdrawn from the American market due to supposedly association with cases of intussusception. Intestinal invagination or intussusception is a condition in which one segment of the intestine invaginates in the adjoining segment. The real incidence of this adverse event is difficult to be evaluated, suggesting a risk of 1 for 10 thousand vaccinated children. Later, some studies indicated that the risk of intussuscption associated to vaccination with RotaShield^®^ was related to the administration of the first dose in children aged ≥90 days. Children aged between 3 and 9 months present increased natural susceptibility to intussusception. Hence, RRV-TV is still licensed in the United States, but its use has been discontinued.^(30)^ RotaShield^®^ has been recently assessed in clinical trials in African countries (South Africa and Ghana), after modifications in the vaccination schedule.^(31)^ The experience with RotaShield^®^ made the Global Advisory Committee on Vaccine Safety (GACVS) orient towards inclusion of assessment of risk of intussusception in the development of new vaccines against RVA.^(30)^

Currently, there are two vaccines licensed and recommended by the World Health Organization (WHO) that proved safe and efficient. RotaTeq^®^ (RV5) (Merck & Co. Inc., West Point, Pennsylvania) is an oral pentavalent attenuated vaccine, which contains five human and bovine viral genotypes - G1 to G4 + P[8] – combined by reassortants. RotaTeq^®^ should be administered in three doses to children aged between 1 and 8 months; in that, the first dose between 6 and 12 weeks of life, the second, between 4 and 10 weeks after the first dose, and the third, from 4 to 10 weeks after the second dose; however, the last dose should be given at most at 32 weeks of age.^(32)^

Rotarix^®^ (RIX_4414_) (GlaxoSmithKline Biologicals, Rixensart, Belgium) is an oral monovalent attenuated vaccine, composed of the G1P1A[8] strain that represents the most common human genotype of RVA. It is recommended to administer two doses of Rotarix^®^ - the first at 2 months and the second at 4 months of age.^(30)^ In Brazil, the vaccine Rotarix^®^ was included in the National Immunization Program, in 2006. RotaTeq^®^ is also licensed in the country but it is available only in private vaccination services.^(33)^

In 2000-2001, China introduced in its National Immunization Program the oral vaccine LLR (attenuated strain of G10P[12]), derived from isolated lamb RVA. Nonetheless, the efficacy of this vaccine is unknown since it was not tested against placebo in phase III clinical tests.^(30)^

Rotavac^®^, a new oral RVA vaccine was launched in India, in 2014, and has been licensed exclusively in that country, so far. Rotavac^®^ was developed and manufactured in India, and is an oral monovalent G9P[11] (11E6) vaccine, derived from a naturally attenuated human neonatal strain, which contains bovine strain segment (natural human-bovine recombinant).^(34)^

### Diagnosis of the Group A rotavirus

The most widely used method for laboratory diagnosis is the enzyme immunoassay (EIA or ELISA), which detects the RVA group antigen (VP6) directly on stools. The latex agglutination tests are often used at hospitals for low cost, fast and easy procedures, but their sensitivity and specificity are lower as compared to EIAs. Reverse transcription followed by nested/multiplex polymerase chain reaction (RT-PCR) has the advantage of making molecular diagnosis and typing of RVA simultaneously. The genetic sequencing methods - microarray and real-time PCR (RT-qPCR) - are very sensitive and capable of discriminating mixed RVA infections, and have been successfully developed and employed in diagnosis of RVA.^(35-37)^

### Epidemiology of Group A rotavirus in humans in pre- and post-vaccination eras

Group A rotavirus infection is ubiquous and affects humans and animals of all ages. Approximately 95% of children all over the world present RVA infection when aged between 3 and 5 years. Group A rotavirus is the most common cause of diarrhea in children aged <3 years worldwide, and the peak of incidence is in children aged 4 to 36 months, who are more susceptible to hospitalizations, primarily due to dehydration, and the economic impact is quite significant.

Group A rotavirus is the main cause of morbidity and mortality associated with diarrhea in Latin America and the Caribean, where it is estimated to account for 8,000 deaths in children <5 years, per year.^(38)^ The symptomatic infections are primarily observed in children aged 6 months to 2 years. Group A rotavirus is one of the main pathogens involved in nosocomial diarrhea outbreaks in day care centers and kindergardens.^(39,40)^ Infections in neonates are frequently asymptomatic, probably due to passive transfer of maternal antibodies. Symptomatic infections in neonates are generally associated to non-usual strains of RVA.^(41)^ Adults infected by RVA are often asymptomatic or present subclinical infections due to presence of neutralizing antibodies previously adquired during natural primary and/or secondary infections. Group A rotavirus infection in adolescents and adults is usually associated with sporadic outbreaks in closed environments, such as schools, offices or hospitals. Group A rotavirus can also infect parents of sick children, travelers, as well as immunocompromised and elderly individuals.^(23,41)^

A fundamental characteristic in epidemiology of RVA is its markedly seasonal pattern. In temperate climates, RVA infection occurs in the coldest and driest months of the year (fall and winter), whereas in tropical climates, the rates tend to be equally distributed throughout the year. The seasonality of RVA varies in Brazil – there is an increased incidence from May to September (coldest and driest period) in the Central, South and Southeast regions. In the North and Northeast, the occurrence of RVA is uniformly distributed during the year.^(41-43)^

The epidemiological surveillance of RVA has been conducted in Brazil since the 1980’s.^(39,44)^ The surveillance systems were intensified in Brazil and other countries after RVA vaccine implementation to document effectiveness of the immunization programs. Changes in epidemiology of the disease caused by RVA are expected in the post-vaccine era.^(40,45)^ The tendency of RVA infecting older children (aged 6 to 10 years) after introduction of the vaccine was reported in the United States and Brazil. The explanation would be indirect protection of non-vaccinated children, related to reduced transmission of RVA in the community (“herd immunity”), resulting in a cohort of susceptible individuals comprising older children who have not been exposed to natural RVA infection previously. Such phenomenon had not been observed in the clinical trials of vaccines currently licensed in the market. However, one cannot predict if RVA transmission will persist in the older age groups, even if vaccination coverage increases among younger children. Studies on implementation of vaccines also identified changes regarding seasonality of RVA infections. The United States, Belgium and Brazil demonstrated a delay of one to two months in beginning of RVA infection season after introducing the vaccine.^(33,46,47)^ The indirect benefits and/or losses caused by RVA vaccination should be further analyzed.

### Distribution of Group A rotavirus strains in humans in the pre- and post-vaccination eras

The differentiation of RVA strains determined by the combination of G and P types is widely used in epidemiological studies,^(1)^ and the genotypes are particularly distributed among several animal species.^(6,9)^ Thanks to the segmented feature of the RVA genoma, the genes that encode for VP7 and VP4 can, in theory, segregate in an independent manner, resulting in a large diversity of strains. The combinations G1P[8], G2P[4], G3P[8] and G4P[8] are historically considered the most prevalent in humans, worldwide (Table 1).^(14)^ Nonetheless, in the past 10 years, G9 strains have been often detected, and they are generally associated to P[8]. Hence, G9P[8] currently ranks fifth among the most prevalent genotypes in humans (Table 1).^(14,45,48,49)^ Recently, G12P[8] was recognized as an emergent genotype, and it seems to expand all over the world, including Brazil.^(50,51)^

**Table 1:** Summary of distribution of Group A rotavirus strains that usually infect humans and animals

Animal	G Genotype	P Genotype
Human	G1; G2; G3; G4; 9	P[4]; P[8]
Equine	G3; G14	P[12]
Bovine	G6; G8; G10	P[1]; P[5]; P[11]
Swine	G9	P[23]
Ovine	G3; G6; G10	P[1]; P[11]; P[14]
Caprine	G6	P[5]
Canine	G3	P[3]; P[5]
Feline	G3	P[3]; P[9]
Leporide (rabbit)	G3	P[14]; P[22]
Birds	G6; G7; G10; G22; G23	P[37]
Procyonide (raccoon)	G8	P[9]
Quirópteros (bat)	G3; G25	P[3]; P[6]
Ursine (giant panda)	G1	P[7]
Wild swine (wild boar)	G4; G9	P[6]; P[13]; P[23]
Artiodactyla (vicuna and giraffe)	G8; G10	P[11]; P[14]

In developed countries, G1P[8], G2P[4], G3P[8], G4P[8] and G9P[8] strains (the most frequent strains worldwide) are detected in approximately 100% of RVA infections. In developing countries, besides the strains more often detected, some less common combinations of RVA have also been identified, showing wide variation from one region to another. A surveillance program conducted by the WHO, in 2010, demonstrated the most predominant uncommon strains were G12P[8] and G12P[6], in Southeast Asia; G2P[6], G3P[6] and G1P[6] in Sub-Saharian Africa; G1P[4] and G2P[8] in Western Pacific; and G9P[4] in the Americas.^(48,52)^

Several studies indicate the predominance of the RVA genotypes in the human population varies along time. A certain dominant strain for one or two years can be replaced by another emerging strain.^(49,53)^ Other strains may be periodically or locally important, such as G5P[8] in Brazil, during the 1980’s, and G8, in Africa.^(48,54)^ The epidemiological basis of the genotypical cycling observed in RVA is still not clear. It is believed that the seasonal change of RVA strains could be a mechanism used by the virus to escape from group immunity acquired in previous infections, thus persisting in the human population.^(53)^

As from 2007, an increased detection of G2P[4] has been observed in Brazil. Some authors suggested this prevalence would be associated to vaccine pressure, and the introduction of a monovalent G1P[8] vaccine could have created conditions for the G2P[4] strain acquiring advantages over the other strains (that share the P[8] genotype) when competing for infection of susceptible hosts.^(33,55)^ However, other common (G1P[8], G3P[8] or G9P[8]) strains continued to circulate marginally along time,^(33)^ as observed in surveillance of RVA strains in Australia.^(56)^ The emergence of G2P[4], in 2007, was also reported in countries that introduced the pentavalent vaccine Rotateq^®^ in their vaccine calendars, like Australia and Nicaragua,^(56,57)^ and in non-vaccinated populations (such as in Portugal, Argentine and Paraguay).^(58)^

The periodicity in circulation of RVA genotypes is a documented fact. In Brazil, G2P[4] presents a cyclical pattern of approximately 10 years,^(49)^ and this should be considered an alternative explanation for increased detection of G2P[4] after 2007.^(40)^ The monitoring of circulating RVA strains in adults was carried out in Brazil, aiming to clarify the supposed vaccine pressure on the pediatric population. In this study, a high prevalence of G2P[4] was also observed, suggesting this emergence probably follows a world trend dictated by oscillatory fluctuations of RVA genotypes, which apparently is not related to vaccination.^(59)^

With the purpose of better understanding changes in the distribuition of RVA strains after the introduction of Rotarix^®^ in Brazil, several analyses of time series of the genotypes detected were performed between 2006 and 2014. This approach addressed important time changes in the country. The predominant and sustained circulation of G2P[4] strains was observed along consecutive years after introduction of Rotarix^®^ (2006 to 2010), but its detection gradually decreased as from 2011. As expected, due to high rates of vaccine coverage in the country, the G1P[8] strains were detected in low prevalence. The frequency of detection of G9P[8] strains abruptly decreased in the following two years, after introduction of Rotarix^®^, resurging as the dominant genotype in 2011, and, it soon dropped again, in 2012. In contrast, the G3P[8] strain, which was rarely detected in the South, Southeast and Central Western Regions of Brazil, for 3 consecutive years (2006 to 2008), progressively had its detection rate risen in 2009, 2010 and 2011, achieving a peak of prevalence in 2012 and 2013. G12P[8] was first detected in the 2008 and 2009 seasons, with a low prevalence. Between 2011 and 2012, the G12P[8] strains had a gradually increased circulation. In 2013, the detection of G12P[8] markedly diminished, and it suddenly reemerged as the most prevalent genotype in 2014.^(33,51,60-62)^ By and large these studies demonstrated a fast change in the distribution pattern of prevalent RVA genotypes circulating in Brazil after the introduction of the vaccine, stressing that continuous surveillance of RVA genotypes is fundamental to assess the impact and success of vaccination.

The emergence of G12P[8] genotype as an epidemiologically important strain all over the world leads to new concerns about development and monitoring of vaccines against RVA, regarding the capacity to induce heterotypical protection against these G12 strains. The efficacy assays of two licensed RVA vaccines (Rotarix^®^ and RotaTeq^®^) were performed with the most prevalent G genotypes (G1, G2, G3, G4 and G9), and the data on efficacy against G12 are very limited.^(63)^ The presence of VP4 P[8] protein in G12 strains suggests both vaccines can act efficiently against the emergence of G12P[8] strains.^(64)^

Therefore the sentinel laboratories worldwide monitor the circulation of RVA strains after introduction of a vaccine, aiming to detect rare or uncommon G(s) and P(s) types not included in the vaccines sold and/or candidate vaccines.^(40)^ Despite the possibility of emergence of new RVA strains, vaccination with Rotarix^®^ and/or RotaTeq^®^ significantly reduced the incidence of diarrheal disease caused by RVA, maintaining levels lower than observed in the pre-vaccine period.^(64)^

### Distribution of Group A rotavirus strains in animals

There is a great variety of circulating RVA strains in animals. The G3, G5, G10, G14 and P[12] genotypes are commonly found in equines.^(9,65)^ The RVA that infect bovines are related to G1, G6-G8, G10, G11, G15, G18 and G21, and P[1], P[5], P[11], P[14], P[17], P[21] and P[29] genotypes; in that, G6, G8 and G10 are associated to P[5], P[11] and P[1], which are the most prevalent.^(6,9,66)^ G6 is often detected in livestock and G10, in dairy cattle.^(67)^ It is very interessing to note that, likewise in humans, cyclic variations were also observed in the genotypes detected in bovines in Japan: G10 (1995), G8 (1996) and G6 (1997) (Table 1).^(68)^

Several genotypes have already been described in pigs: G1-G6, G8-G12, P[1], P[5-8], P[11], P[13], P[19], P[21-27] and P[32].^(69)^ The RVA strains isolated in lamb belong to the G1, G3, G5, G6, G8, G9 and G10 genotypes, and the G3, G6 and G10 genotypes are often associated to P types belonging to P[1], P[11] and P[14].^(6)^ In caprines, the most common genotype is G6P[5],^(6)^ but other strains have already been described: G3P[3] in South Korea, G6P[14] in South Africa, and G6P[1] in Italy (Table 1).^(70)^

In domestic animals, G3 is the most prevalent genotype, and it is usually associated to P[3] and P[5] in dogs, and a P[3] and P[9] in cats.^(6,71)^ G3 is also often detected in rabbits, but associated to P[22] and P[14].^(72)^ There is limited information about circulanting genotypes in birds, and some studies reported detection of G7, G23, G22, G6, G10 and P[37].^(8,20)^ Nonetheless, the RVA detected in birds seems to be genetically heterogeneous when compared to that in mammals (Table 1).^(20)^

The RVA genotypes circulating in wild animals are virtually unknown. There are reports of detection of G3P[9] in racoons (*Nyctereutes larvata*) and masked palm civets (*Paguma larvata*) in Japan;^(7)^ G25P[6] in fruit bats (*Eidolon helvum*) in Kenya;^(73)^ G8P[14] in vicunas (*Vicugna vicugna*) in Argentina;^(66)^ G1P[7] in a giant panda in China;^(74)^ G3P[3] in lesser horseshoe bat (*Rhinolophus hipposideros*) in China;^(75)^ G9P[23], G4P[23], G9P[13] and G4P[6] in wild boar (*Sus scrofa*) in Japan^(76)^ and G10P[11] in a giraffe in the Dublin Zoo, in Ireland.^(77)^ However, it is still not possible to conclude if these animals are commonly infected by these genotypes, or if infections result from interespecies transmissions and/or gene reassortments (Table 1).

### Group A rotavirus transmission

Group A rotavirus infections are mainly acquired by fecal-oral route, including fomites and person-to-person contact with contaminated objects. Group A rotavirus transmission through consumption of contaminated water or foods was reported, but rarely occurs. Although RVA were detected in urine samples and in the upper respiratory tract, these body fluids are not often associated to RVA transmission.^(1)^

### Interspecies transmission and zoonotic potential of Group A rotavirus

The capacity of RVA transmission among mammal species was demonstrated in the 1980’s.^(6)^ Castrucci et al.^(78)^ demonstrated that calves were susceptible to RVA infection of rabbits, which, in turn, had been infected with bovine RVA. Calves are equally susceptible to RVA infection of simian, swine or leporid origin.^(78)^ Bovine RVA excretion was detected in dogs and cats, and RVA transmission from mammals to birds has already been documented.^(79,80)^

The advent of molecular biology enabled identification of animal RVA strains infecting humans in different parts of the world, including Brazil.^(81)^ The complete genoma sequence analysis of human RVA strains belonging to G3P[3] (Ro1845 and HCR 3A) genotype revealed that both strains are closely related to canine (CU-1; K9 and A79-10) and feline RVA (Cat97).^(71)^ The equine RVA seems to have a very close genetic relation with human and swine RVA.^(82)^ The G3 (common in cats, dogs, pigs and horses), G5 (common in pigs and horses), G6, G8 and G10 (common in cattle) and G9 (common in pigs and sheep) RVA strains were identified in human populations in different parts of the world. The swine G4, G5, G6 and G8 genotypes were found circulating in humans, calves and camels.^(6,9)^ These data suggested pigs might be the main RVA reservoir and source of emerging strains, in humans and other animals.^(9)^ Based on these facts, the RVA should be considered as potential zoonotic pathogens.

The main zoonotic transmission means is intimal contact between humans and animals. The risk of such transmission is also present in contamination of water reservoirs or foods by feces of infected animals.^(6,9)^ Nevertheless, the studies on RVA zoonoses have an important limitation: there is no epidemiological relation between human and animal cases. Hence, the study of the zoonotic event of a particular RVA strain is confirmed only based on filogenetic evidence and data available on the frequency of detection of a specific genotype, in a particular host.^(81)^

### Genetic diversity and evolution of the Group A rotavirus

The RVA diversify and evolve by means of two main mechanisms. The first is accumulation of point mutations, which will originate the genetic lineages and lead to emergence of mutants able to escape the previously existing antibodies. The second mechanism is gene shift, changing genetic material by genetic reassortants during infection of a single cell by two or more different types of RVA.^(1,83)^

Reassortments between the animal and human RVA may occur, generating chimeric viruses containg genomic segments of both RVA. The RVA is said to cross the interspecies barrier, but when doing it, the virus is not able to infect and disseminate the new host in an efficient manner. However, when adquiring human segments, these chimeric viruses would increase their chances of efficiently propagating among the human population. Hence zoonotic transmission and gene segment reassortment between animal and human RVA do contribute to increased diversity of strains that infect humans.^(81)^ A crucial factor for RVA reassortant generation is the high frequency of coinfection. In developing countries the coinfection rate is higher (approximately 20%) than in developed ones (roughly 5%). This fact can also explain why the frequency of detection of atypical strains is higher in developing countries.^(48)^

There is increasing evidence all over the world that uncommon G and/or P tyes may become epidemiologically important.^(84)^ Nonetheless, it is difficult to predict which strains will be able to disseminate globally. The G9P[8] strain is a recent example of this dissemination, since it was first considered rare and today it takes a dominant position among the circulating strains worldwide.^(48)^ Some reports have recently showed an increased detection of G12 genotype, indicating this will probably be the next strain to become globally dominant.^(48,50)^ Filogenetic evidence indicate these two strains - G9 and G12 - were probably swine and started infecting humans based on gene reassortments.^(9)^

## CONCLUSION

Group A rotavirus is the main etiologic agent of acute diarrheal disease in children worldwide. Nevertheless, a significant reduction in cases of disease group-A-rotavirus-associated gastroenteritis was observed after vaccine introduction in many countries, including Brazil. Today it is important to expand monitoring of acute diarrheal disease related to other pathogens (viral or not) in children aged under 5 years. The post-vaccine era led to a new epidemiological scenario regarding infections caused by Group A rotavirus, and the continued surveillance of genotypes is crucial to identify the emerging strains, as well as to assess vaccine efficacy in differents regions of the world. There are robust interactions between animal and human group A rotavirus, but the zoonotic studies are limited by scarce availability of genome sequences of animal RVA. The simultaneous surveillance of RVA infections in animals (including wild species) and humans, and the accumulation of nucleotide sequences from animal strains are vital to understand the ecology, epidemiology and evolution of such viruses.
